# Birds With Distinct Ecological Traits Show Varied Haemoglobin Adaptations Along Elevation Gradients

**DOI:** 10.1002/ece3.71203

**Published:** 2025-04-09

**Authors:** Zamekile D. Bhembe, Sara Padidar, Kat Bebbington, Sjouke A. Kingma, Ara Monadjem

**Affiliations:** ^1^ Department of Biological Sciences University of Eswatini Kwaluseni Eswatini; ^2^ Behavioural Ecology Group, Department of Animal Sciences Wageningen University and Research Wageningen the Netherlands; ^3^ Mammal Research Institute, Department of Zoology & Entomology University of Pretoria Hatfield South Africa

**Keywords:** ecological traits, elevation gradient, haemoglobin concentration, hypoxia

## Abstract

Ecological systems are highly dynamic, with organisms continually adapting to various environmental stressors along natural gradients. Birds along elevation gradients serve as excellent models for examining physiological adaptations, such as elevated haemoglobin concentrations at high altitudes due to lower oxygen availability. This study aimed to examine how various ecological traits influence the haemoglobin concentration responses of multiple bird taxa to an elevation gradient. We measured haemoglobin concentration in 920 birds of 133 species at six sites representing an elevation gradient spanning from 60 to 1,600 m above sea level. Using MCMC Bayesian mixed models, we identified important ecological determinants of haemoglobin concentration and further ran separate models to test whether haemoglobin concentration responses to elevation differ between various functional groups of birds. Our results showed that haemoglobin concentration increased significantly with elevation and was strongly influenced by wing morphology, body mass, season, and primary lifestyle. The rate of increase with elevation varied by lifestyle: terrestrial and perching birds exhibited a steeper increase in haemoglobin concentration with elevation, while aerial birds also increased haemoglobin but at a more gradual rate. However, the remaining traits did not alter how species respond to hypoxia; for example, birds increased haemoglobin at the same rate in both the dry and wet seasons, meaning seasonal changes did not strongly impact elevation‐driven haemoglobin adjustments. Elevation is the primary driver of variation, while lifestyle influences baseline levels rather than the rate of change. Despite differences in lifestyle‐driven oxygen demands, birds exhibit a similar haemoglobin response to hypoxia at moderate elevations, where adjustments remain within physiological limits, indicating that hypoxia‐driven haemoglobin adjustments occur independently of baseline oxygen demands. These findings demonstrate how birds regulate oxygen transport relative to ecological constraints, providing insights into their physiological flexibility across environmental gradients.

## Graphical abstract

Birds along elevation gradients provide unique insights into physiological adaptations to hypoxia. This study examined how ecological traits influence haemoglobin concentration in 920 birds across 133 species sampled along a 60–1600 m elevation gradient. Our findings show that haemoglobin increased with elevation, with a steeper rise in terrestrial and perching birds than in aerial species. Elevation was the main driver, while lifestyle affected baseline levels but not the rate of change along an elevation gradient.

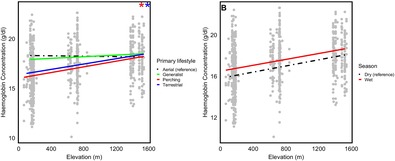

## 1 Introduction

Ecological systems are highly dynamic, and living organisms continually interact with their environment, adapting to diverse environmental stressors. Abiotic factors such as rainfall, temperature and oxygen availability change predictably with elevation, shaping species distributions and physiological adaptations (Zamora‐Vilchis et al. 2012, Chamberlain et al. 2016). As a result, elevation gradients have been successfully used as a natural experiment to examine the adaptations of species to their environment (Storz 2007, 2021; González‐Morales et al. 2023). One of the most critical stressors associated with elevation is hypoxia, a condition caused by declining barometric pressure at higher altitudes, which reduces atmospheric oxygen partial pressure (Ramirez et al. 2007; Farrell and Richards 2009). Hypoxia poses a significant physiological challenge for vertebrates at higher elevations, particularly those with high metabolic demands, as oxygen is essential for aerobic performance and energy balance of living organisms (Sharma et al. 2022; Burnett and Stickle 2011).

To cope with hypoxia, high‐altitude species adopt different structural and functional adaptations, such as increased lung capacity, enhanced capillary density, and modifications to oxygen transport systems (Storz and Scott 2019; Storz et al. 2010; Farrell and Richards 2009). These adaptations are well documented in species resident at extreme elevations, such as the Himalayas and the Andes mountains (Butler 2010; Laguë 2017), where vertebrates have evolved specialised adaptation traits that help them survive chronic oxygen deprivation. However, species inhabiting less extreme elevations also face fluctuating environmental conditions that create such physiological challenges, and the ability of species to move between low‐ and mid‐elevation zones over short geographic distances exposes individuals to diverse environmental conditions along elevation gradients (Dubay and Witt 2014). Although hypoxia is less pronounced at moderate elevations, its effects remain ecologically significant, particularly for species that are not permanent high‐altitude residents. Short‐term exposure may favour reversible physiological adjustments rather than permanent genetic adaptations observed in high‐altitude resident species (Storz and Scott 2019).

One of the most critical adaptations to oxygen‐related stressors is the regulation of blood haemoglobin concentration to cope with varying oxygen availability (Minias 2020; Minias et al. 2013). Haemoglobin is a crucial component of the blood's oxygen transport system, binding oxygen in the lungs and delivering it to tissues for metabolic processes; therefore, it is a key indicator of oxygen‐carrying capacity (Seibel and Deutsch 2020; Nikinmaa 2001). Vertebrates increase haemoglobin concentration to enhance oxygen transport efficiency, improving survival in low‐oxygen environments (Seibel and Deutsch 2020; Storz et al. 2010). Haemoglobin increase is a well‐established physiological response to hypoxia and has been widely documented across various vertebrates, including birds (Minias 2020; Storz et al. 2009). Birds are among the most widely distributed vertebrates in the world, surviving in a wide range of habitats from dense tropical forests to the highest mountain peaks (Callaghan et al. 2021). Their ability to occupy such diverse environments results from a complex interplay of physiological mechanisms that allow them to thrive in diverse and often challenging environments (Lees et al. 2022; Callaghan et al. 2004). While the general trend of increased haemoglobin concentration with elevation is widely recognised in birds (Minias 2020; Siebenmann et al. 2023), the role of species ecology in shaping this response is often overlooked. Yet, interspecific variation in haemoglobin responses suggests that there are species‐specific responses to hypoxia resulting from multiple interacting factors.

Birds exhibit diverse ecological and functional traits, each of which imposes distinct physiological demands. This means that birds adapted to specific traits such as flight strategy and foraging behaviour will have different haemoglobin concentrations based on their oxygen needs. For example, birds that engage in energetically costly activities such as aerial foraging, breeding and territoriality require significantly more energy compared to less demanding strategies like ground foraging or nonterritoriality (Sibly et al. 2012; Ruiz et al. 1995). Studies have reported that migratory bird species that traverse large geographical ranges have higher haemoglobin concentrations compared to nonmigratory species in the same area, which is an adaptation to enhance their migration abilities (Barve et al. 2016; Yap et al. 2019). These high energy demands translate into a greater need for oxygen, which in turn drives physiological adaptations like increasing haemoglobin concentration to sustain such lifestyles. This raises the question of whether such species will still be able to further increase haemoglobin levels under hypoxic conditions without negating the benefits, or if their haemoglobin levels are already optimised for peak oxygen demand. It is therefore reasonable to expect that different functional groups would exhibit distinct physiological adaptations to hypoxia.

Seasonal variability adds another layer of complexity to species' oxygen demands. Temperature fluctuations, food availability and reproductive cycles influence the metabolic performance of bird species (Renthlei et al. 2022; Swanson 2010). Birds experience fluctuating energy demands throughout their life‐history stages, with peaks during energetically demanding activities associated with breeding such as nest building, egg laying, brooding and caring for their young (Pacioni et al. 2023; McKechnie and Swanson 2010). These seasonal changes in energy demands are particularly important in African savannah ecosystems; seasonal changes drive distinct variations in habitat structures and resource availability (Wei and Barros 2021; Zwarts et al. 2023). During the wet season, which coincides with the breeding season for many bird species (Dinesen et al. 2022; Zwarts et al. 2023), energy‐intensive activities associated with reproduction may intensify the metabolic challenges imposed by hypoxia, which have physiological consequences (Williams 2012). Understanding how birds adjust their oxygen transport mechanisms in response to both elevational constraints and seasonal metabolic shifts will provide critical insights into their capacity for physiological flexibility versus long‐term adaptation.

In addition, traits such as body size may also influence haemoglobin variation among bird species. Larger birds have higher absolute oxygen consumption, which may drive increases in haemoglobin levels to meet their greater metabolic demands associated with their body size (Lill et al. 2013). However, some species may maintain consistently high haemoglobin levels not solely due to their size but because of their adaptations for efficient long‐distance dispersal. Flight efficiency, often assessed through wing morphology (e.g., hand–wing index), plays a crucial role in oxygen transport (Weeks et al. 2022). Species with a higher hand–wing index (longer, more pointed wings) are adapted for sustained flight and long‐distance movement, requiring consistently elevated haemoglobin levels to meet the demands of prolonged aerobic activity. In contrast, species with a lower hand–wing index (shorter, rounder wings) are best adapted for manoeuvring over endurance, which may reduce their reliance on elevated haemoglobin concentrations for efficient flight (Claramunt 2021; Sheard et al. 2020). Incorporating these traits into analyses of haemoglobin responses ensures a more accurate interpretation of how different species regulate oxygen transport under varying ecological constraints.

The differences in oxygen demand by functional and ecological groups raise important questions about the role of species ecology in the physiological adaptations among bird species. That is, while an increase in haemoglobin concentration with elevation is a general trend and a critical adaptation for survival in hypoxic environments (Minias et al. 2013; Storz et al. 2009), the degree to which these responses are influenced by species specific traits is likely complex and not yet fully understood. Birds along elevation gradients offer a good opportunity to study these determinants of haemoglobin concentration, providing insights into the drivers of physiological diversity among species (Barve et al. 2016).

Research on haemoglobin concentration in tropical birds, particularly in Africa, remains limited. The few African studies that exist are from regions north of the Sahara such as Egypt, where migratory Eurasian ducks have been studied during their overwintering period (Elarabany 2018). Although meta‐analyses and reviews (Minias 2015; Minias 2020; Fair et al. 2007) have synthesised patterns of haematological traits in birds, they do not explicitly test whether species' ecological characteristics influence haemoglobin responses to elevation. Moreover, research has been conducted on montane birds in high‐altitude regions, examining how haemoglobin concentration helps them to cope with hypoxia (Ishtiaq and Barve 2018; Barve et al. 2016); these studies explored haemoglobin variation across species with different migration patterns, which provided important insights into physiological adaptations. However, our study expands on this by incorporating a broader range of ecological variables, not just as a function of migration, and by focusing on an African savannah system, which may exhibit unique physiological responses due to its distinct environmental and ecological factors.

While previous studies have identified various ecological determinants of haemoglobin concentration (Minias 2015; Minias et al. 2014; Kaminski et al. 2014), the interplay between these ecological factors and variation in elevation remains less understood. To address this gap, we explored haemoglobin concentration across a broad range of species along an elevation gradient. The main aim of this study was to examine how various bird traits influence the haemoglobin concentration responses to an elevation gradient across multiple bird taxa while controlling for species phylogeny to ensure that observed differences in haemoglobin concentration reflect adaptive responses to hypoxia rather than inherited traits. To achieve this, we had two main objectives: (1) identify the key ecological determinants of haemoglobin concentration in multiple bird species recorded along an elevation gradient, laying the groundwork for our next objective; (2) to use these ecological predictors to determine whether species with different ecological traits exhibit distinct physiological adjustments to increasing elevation. We hypothesised that energetically demanding ecological traits would significantly influence haemoglobin concentration in birds. We also predicted that birds with energetically demanding ecological traits (e.g., migrants, aerial foragers and territorial birds) will have reduced physiological flexibility to adjust haemoglobin concentration at high elevations, whereas species with lower energy demands will show greater plasticity in response to hypoxia.

## 2 Methods

### 2.1 Study Area and Design

**FIGURE 1:**
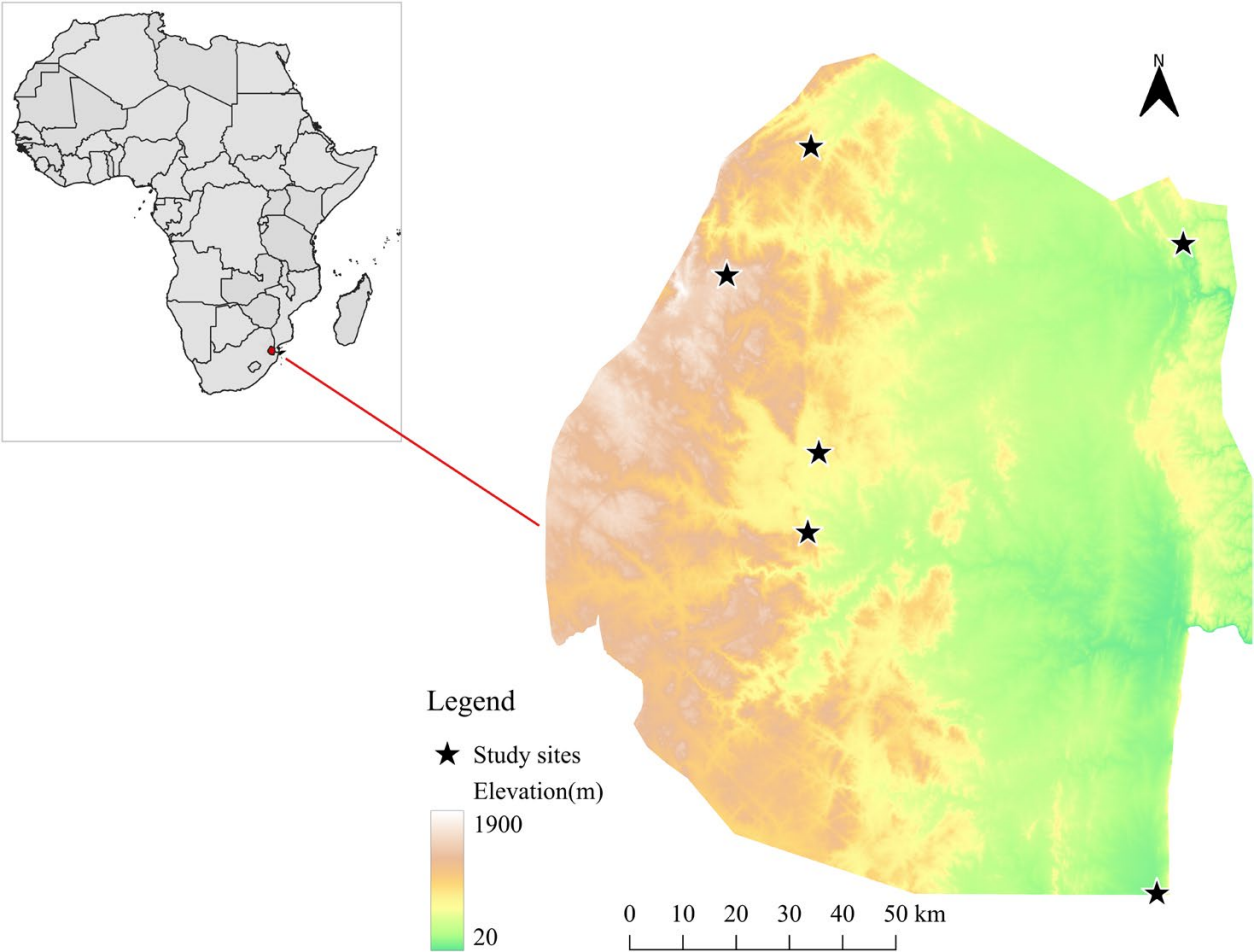
Map of Eswatini showing the elevation gradients of Eswatini and the study sites (black stars). Inset: Map of Africa highlighting Eswatini in red (red arrow pointing to its location). Note that elevation is highly variable over a relatively small geographical scale.

The study was conducted in Eswatini, a small landlocked country in the southern African region, bordered by South Africa and Mozambique. Despite its small size (17,365 km^2^), the country has a great variation in elevation over a relatively small geographic scale, ranging from 21 m above sea level (ASL) in the east to 1862 m ASL in the west (Dlamini and Loffler 2023). The country is situated entirely within the savannah biome, which offers the opportunity to conduct elevational studies within a single biome. In this study, we selected a total of six study locations ranging between 143 and about 1500 m above sea level that spanned the range of elevations present in the country (Figure 1 and Table 1).

**TABLE 1:** Coordinates for each study location and the mean elevation at each site.

Location	Coordinates	Mean ± SD elevation (m a.s.l.)
Royal Jozini Game Reserve	−27.31489 S, 31.9533 E	143 ± 0
Mbuluzi Game Reserve	−26.14116 S, 32.6072 E	168 ± 17
Matsapha, Kwaluseni	−26.4776 S, 31.30721 E	649 ± 2
Wild Horizons Wildlife Sanctuary	−26.3347 S, 31.29002 E	705 ± 42
Phophonyane Falls Nature Reserve	−25.89645 S, 31.296 E	932 ± 84
Malolotja Nature Reserve	−26.1531 S, 31.10291 E	1452 ± 50

### 2.2 Data Collection

We collected data between October 2021 and December 2023, conducting a total of four sampling sessions per site across consecutive dry (April to September) and wet (October to March) seasons (two sessions per season). We spent at least 5 days sampling at each site per session, aiming to maximize the total number of birds captured at each site.

We captured birds through passive mist netting, setting up mist nets before dawn to capture the early morning peak of bird activity. We closed the nets during the day when temperatures rose and bird activity decreased, then reopened them in the afternoon when bird activity increased. After capturing birds, we placed them in individual cloth bags and noted the time of day at which each bird was caught. We then identified birds to species (Roberts et al. 2005) using the taxonomy of BirdLife International (BirdLife International 2020), and measured a standard set of biometrics including tarsus length (to the nearest 0.1 mm) and wing length (to the nearest 0.5 mm), and weighed birds (body mass to the nearest 0.1 g) using a Pesola spring scale.

To measure the haemoglobin concentration, we bled each bird from the ulnar vein using a disposable hypodermic needle, taking approximately 10 μL blood samples from each bird into a disposable HemoCue cuvette (HemoCue AB, Angelholm, Sweden) and analysing them using a HemoCue Hb 201+ photometer (HemoCue AB, Angelholm, Sweden), which provided an instant quantification of haemoglobin concentration (g/L). We then fitted birds with a uniquely numbered aluminium ring (SAFRING, South Africa) to prevent double sampling of recaught birds. After collecting all measurements and samples in the field, we released birds at the location of capture.

### 2.3 Ecological Variables

Our study included six ecological traits (elevation, season, territoriality, migration, and primary lifestyle) along with one functional trait (hand–wing index) in line with (Violle et al. 2007). We recorded elevation at every netting site using a handheld GPS (eTrex 30×, Garmin). We chose ecological variables that either (a) have been previously shown to influence haemoglobin concentration in multiple taxa or (b) are important in terms of energy consumption, which may potentially affect haemoglobin concentration. We extracted data on territoriality behaviour from a published dataset (Sheard et al. 2020) and classified each species as nonterritorial, weakly territorial or strongly territorial. The rest of the traits were extracted from the AVONET database set (Tobias et al. 2022). We classified species by four ecological variables from the AVONET database as follows: Migration (migratory for long‐distance migrants and sedentary for all others, including partial migrants, nonmigrants and nomads), Primary lifestyle (aerial, terrestrial, perching or generalist for predominant foraging lifestyle), and one functional trait, hand–wing index (Kipp's distance as a proxy for wing aspect ratio, related to dispersal ability) (Tobias et al. 2022). We describe full details of all variables in Table S1.

**Table:** 

Scale of inference	Scale at which the factor of interest is applied	Number of replicates at the appropriate scale
Elevation	Location	6
Ecological group	Species	Varies by ecological group, pooled across locations

### 2.4 Replication Statement

#### 2.4.1 Data Analysis

We tested the relationship between haemoglobin concentration and ecological variables using phylogenetically informed Markov Chain Monte Carlo (MCMC) Bayesian mixed models (PGLMMs) implemented in the MCMCglmm package (Hadfield 2010) in R version 4.3.1 (R Core Team 2018). By incorporating phylogenetic control in our analysis, we ensured that the observed relationships between haemoglobin concentration, ecological traits and elevation were not solely driven by shared evolutionary history but rather reflected genuine physiological responses. Prior to analysis, we screened the dataset for outliers using Cleveland dot plots. Of the 926 birds sampled, six individuals with exceptionally low or high haemoglobin concentrations were identified as outliers and removed from further analysis.

#### 2.4.2 Phylogenetic Tree Construction

To account for evolutionary relationships among species, we constructed a phylogenetic tree based on molecular data obtained from BirdTree.org (Jetz et al. 2012) using the Hackett backbone. We downloaded a posterior distribution of 2000 ultrametric trees and generated a Maximum Clade Credibility (MCC) tree. The MCC tree was used in all subsequent phylogenetic analyses, with modified versions pruned as necessary to retain only the relevant taxa for each analysis.

#### 2.4.3 Identification of Ecological Determinants

To identify key determinants of haemoglobin concentration while accounting for species' evolutionary relationships, we used data from all captured birds (*n* = 920). Haemoglobin concentration was modelled as a Gaussian response variable, with species phylogeny included as a random effect via an inverse variance–covariance matrix derived from the MCC tree, assuming a Brownian motion model of evolution. This approach allowed us to simultaneously estimate variation among taxa while controlling for phylogenetic relationships and accommodating repeated measures within species in our dataset.

We included seven (7) predictor variables: elevation (m), log‐transformed body mass, and season (dry/wet), which were directly observed, along with hand–wing index, migratory status, territoriality and primary lifestyle, which we obtained from other sources (see Table S1). All continuous predictors were standardised (mean = 0, SD = 1) using the *scale()* function in R to improve model convergence and comparability (Zipkin et al. 2021; Harrison et al. 2018). To obtain the most parsimonious model, we first ran the global model then sequentially removed highly insignificant predictors (*p* > 0.1) and reran the model. However, the Deviance Information Criterion (DIC) indicated no significant difference between the global and reduced models, so we retained the global model for interpretation. To assess the degree to which haemoglobin variation is influenced by shared evolutionary history, we estimated the phylogenetic signal in haemoglobin concentration using Pagel's λ within the PGLMM framework based on the global model. Lastly, we conducted post hoc pairwise comparisons of significant categorical predictors.

To ensure model reliability, we checked for model convergence by visually inspecting trace plots and assessed multicollinearity in predictor variables by calculating variance inflation factors (VIF) using the ‘vif’ function from the R package *car*. All VIF values were below 3 (VIF < 3), confirming no significant collinearity between predictor variables (Dormann et al. 2013).

#### 2.4.4 Consistency of Response Analysis

To assess whether birds with different ecological strategies exhibit distinct haemoglobin responses to elevation, we tested for interaction effects between elevation and the significant ecological predictors identified in the first analysis. To ensure robust comparisons, we selected only bird families in which individuals were sampled across the entire elevation gradient to avoid comparing species restricted to low or high elevations, focusing instead on the representation of families across the entire gradient. In total, 22 of 43 families (751 individuals) met this criterion and were included in the analysis. Using the significant variables identified earlier, we ran separate PGLMMs modelling interactions between elevation and each significant ecological variable (e.g., elevation × season), while maintaining species phylogeny as a random effect. We ran a global model and a reduced model as described previously.

## 3 Results

### 3.1 Results Summary

We captured a total of 926 birds belonging to 133 species in 43 families during the two‐year study period. After removal of the 6 outliers, 920 individuals were considered for analysis. The mean haemoglobin concentration across all birds from all elevations was 17.1 g/dL.

### 3.2 Identification of Ecological Determinants

**FIGURE 2:**
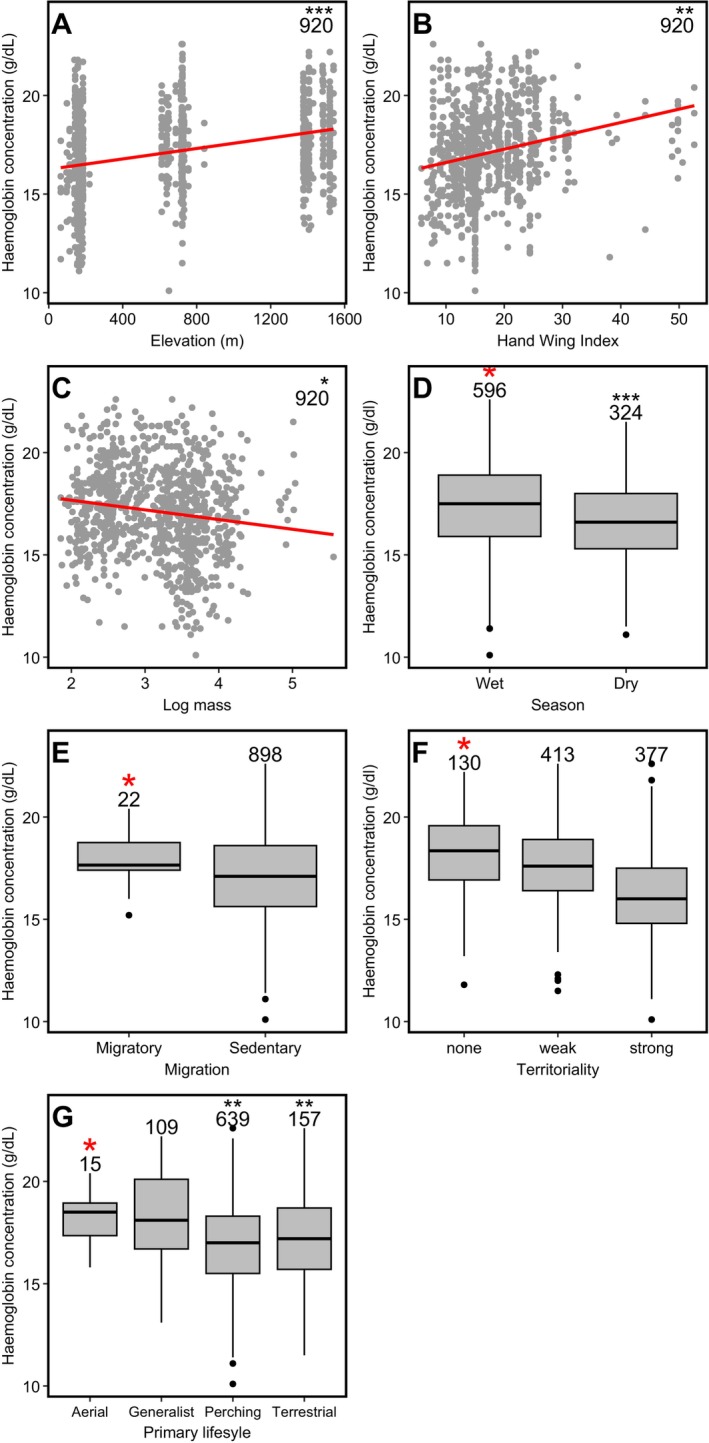
Haemoglobin concentration and ecological variables: Elevation, hand–wing index, log mass, season, migration, territoriality and primary lifestyle in birds recorded along an elevation gradient in Eswatini. Red stars represent reference variables and black stars a significant difference in haemoglobin concentration of each variable compared to the reference variables. The numbers indicate sample sizes.

We found significant effects of elevation, season, body mass, wing morphology and primary lifestyle on haemoglobin concentration, while migration and territoriality showed no strong influence (Table 2). Elevation had a significant positive effect (*β* = 0.53, 95% CI: 0.38, 0.68, *p* < 0.001), indicating an increase in haemoglobin concentration with increasing elevation (Figure 2A). Birds sampled during the wet season had significantly higher haemoglobin concentration compared to those sampled in the dry season (*β* = 0.45, 95% CI: 0.20, 0.71, *p* < 0.001) (Figure 2D). We also found a significant positive relationship between haemoglobin concentration and hand–wing index (*β* = 0.44, 95% CI: 0.10, 0.80, *p* = 0.014) (Figure 2B), while body mass had a significant negative effect on haemoglobin concentration (*β* = −0.26, 95% CI: −0.54, 0.00, *p* = 0.049), suggesting that larger bodied birds had lower haemoglobin concentrations compared to smaller sized birds (Figure 2C).

**TABLE 2:** Bayesian estimates of predictors of haemoglobin concentration in birds in Eswatini. Estimates were derived from a Bayesian generalised linear mixed model (MCMCglmm) accounting for phylogenetic relatedness with a species‐level random effect.

	Estimate	l‐95% CI	u–95% CI	pMCMC	
(Intercept)	14.906	11.904	18.154	< 0.001	***
Elevation	0.530	0.385	0.678	< 0.001	***
Season [Wet]	0.453	0.198	0.708	< 0.001	***
Log mass	−0.258	−0.535	0.002	0.049	*
Hand wing index	0.440	0.100	0.804	0.014	*
Primary lifestyle [Generalist]	2.280	−0.027	4.585	0.066	
Primary lifestyle [Perching]	2.126	−0.231	4.377	0.036	*
Primary lifestyle [Terrestrial]	2.571	0.041	4.821	0.04	*
Migration [Sedentary]	−0.031	−1.457	1.172	0.974	
Territoriality [Strong]	−0.746	−1.617	0.201	0.122	
Territoriality [Weak]	−0.220	−0.982	0.471	0.536	

*Note:* The Estimate column represents the posterior mean effect size, l–95% CI and u–95% CI denote the lower and upper bounds of the 95% credible interval, and pMCMC is the posterior probability of the effect being different from zero. Star signs denote statistical significance, where * ≤ 0.05, ** ≤ 0.01, and *** ≤ 0.001. Reference levels for categorical variables: Season = Dry, Primary lifestyle = Aerial, Migration = Migratory, and Territoriality = None.

Generalist species had a higher haemoglobin concentration compared to aerial foraging birds, but the difference was not statistically significant (*β* = 2.28, 95% CI: −0.03, 4.59, *p* = 0.066). However, perching species (*β* = 2.13, 95% CI: −0.23, 4.38, *p* = 0.036) and terrestrial species (*β* = 2.57, 95% CI: 0.04, 4.82, *p* = 0.040) had significantly lower haemoglobin concentrations compared to aerial foragers (Figure 2G). We also observed no significant difference in haemoglobin concentration between migratory and sedentary species (*β* = −0.03, 95% CI: −1.46, 1.17, *p* = 0.974) (Figure 2E). Similarly, we found no significant influence of territoriality, as neither strongly territorial (*β* = −0.75, 95% CI: −1.62, 0.20, *p* = 0.122) nor weakly territorial species (*β* = −0.22, 95% CI: −0.98, 0.47, *p* = 0.536) differed significantly from nonterritorial species (Figure 2F).

### 3.3 Consistency of Response Analysis

**FIGURE 3:**
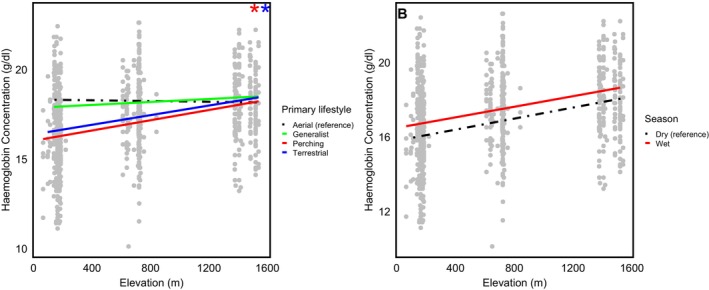
Interaction effects of elevation on haemoglobin concentration across different levels of primary lifestyle and seasons. The plots illustrate how the relationship between elevation and haemoglobin concentration varies among different categories of primary lifestyle (A) trend lines represent primary lifestyle categories (aerial, generalists, perching and terrestrial), while (B) shows seasons (Wet and dry). The black and dashed lines in both graphs represent the reference categories and stars indicate that the line of the same colour is significantly different from the reference significant.

There were significant positive interactions between elevation and primary lifestyle for perching (*β* = 0.38, 95% CI: −1.13, 2.12, *p* = 0.022) and terrestrial species (*β* = 0.35, 95% CI: −1.40, 1.89, *p* = 0.046) (Table 3). This indicates that haemoglobin concentration increased more steeply with elevation in perching and terrestrial birds compared to aerial species (Figure 3A). In contrast, generalist species showed a similar haemoglobin response to elevation as aerial species, with no notable difference in the rate of increase.

**TABLE 3:** Bayesian generalised linear mixed model (MCMCglmm) results illustrating the relationships between elevation and ecological variables for 22 bird families recorded along an elevation gradient, in terms of their haemoglobin concentration (Consistency of response analysis).

	Estimate	l–95% CI	u–95% CI	pMCMC	
(Intercept)	14.508	10.619	18.551	< 0.001	***
Elevation	0.366	−1.158	2.039	0.704	
Season [Wet]	0.438	0.166	0.721	0.002	**
Log mass	−0.329	−0.699	0.064	0.102	
Hand–wing index	0.681	0.242	1.186	0.001	**
Primary lifestyle [Generalist]	2.827	−0.790	6.007	0.104	
Primary.lifestyle [Perching]	2.558	−0.600	5.947	0.023	*
Primary.lifestyle [Terrestrial]	3.023	−0.532	6.479	0.034	*
Elevation: season [Wet]	−0.256	−0.571	0.007	0.074	
Elevation: log mass	0.002	−0.177	0.180	0.98	
Elevation: hand–wing index	−0.045	−0.287	0.236	0.73	
Elevation: primary.lifestyle [Generalist]	0.197	−1.458	1.681	0.79	
Elevation: primary.lifestyle [Perching]	0.376	−1.128	2.122	0.022	*
Elevation: primary.lifestyle [Terrestrial]	0.347	−1.396	1.888	0. 045	*

*Note:* The table presents the posterior mean estimate (Estimate), lower and upper 95% credible intervals (l–95% CI, u–95% CI), and posterior probability (pMCMC). Significant effects are indicated with asterisks (* ≤ 0.05, ** ≤ 0.01, *** ≤ 0.001). Reference levels for categorical variables: Season = Dry, and Primary Lifestyle = Aerial.

No other significant interactions were detected in this analysis. Specifically, the interaction between elevation and season was not significant (*β* = −0.26, 95% CI: −0.57, 0.01, *p* = 0.074) (Table 3), suggesting that seasonal changes had little effect on how birds respond to increasing elevation (Figure 3B).

## 4 Discussion

The aim of this study was to assess the role of species ecology in their response to hypoxia while controlling for phylogenetic effects, where we (1) identified key functional traits influencing haemoglobin concentration in birds and (2) determined whether the response to elevation‐induced hypoxia varies across different functional groups and the role of shifts in energy dynamics resulting from seasonal changes. We sought to understand how varying functional traits might shape the ability of different bird groups to increase haemoglobin concentration with increasing elevation. In the first analysis, primary lifestyle and sampling season were identified as important determinants of haemoglobin concentration in birds. We accounted for phylogenetic relatedness in haemoglobin concentration by estimating Pagel's λ, which had a posterior mean of 0.33. A moderate degree of phylogenetic structuring (Pagel's *λ* = 0.33) indicated that while closely related species tend to have more similar haemoglobin concentrations, other ecological and environmental factors also play a significant role.

In the first analysis, haemoglobin concentration was found to be best explained by elevation, with haemoglobin concentration increasing significantly with it. This pattern reflects a well‐documented physiological adaptation among vertebrates, where reduced oxygen availability at higher altitudes (hypoxia) drives the need to enhance oxygen‐carrying capacity by increasing haemoglobin concentration (Minias 2020; Barve et al. 2016; Lague et al. 2016; Scott 2011; Faraci 1991). This adaptation allows birds to maintain adequate oxygen supply to tissues, enabling effective functioning despite lower oxygen levels at higher elevations. However, the influence of other ecological traits on haemoglobin concentration identified in this study supports our prediction that haemoglobin concentration in birds fluctuates not only in response to hypoxia but also based on species‐specific functional traits (Garcia et al. 2010; Taggart 2016). Variation in energy requirements among ecological groups likely explained the link between functional ecology and haemoglobin concentration, where highly energy‐demanding activities necessitate higher oxygen demand, which affects haemoglobin concentration in birds. This suggests that, regardless of altitude, haemoglobin levels in birds may vary in response to the energetic demands and oxygen requirements associated with their ecological roles.

Sampling season was also a significant predictor of haemoglobin concentration, with higher haemoglobin levels observed during the wet season compared to the dry season. These results support our hypothesis that haemoglobin concentration will be higher in the wet season during which there is peak bird activity in southern African savannah ecosystems. The wet season is aligned with breeding activities in birds such as territorial defence, nest‐building, egg laying, incubation, and rearing young, creating the need to enhance their oxygen‐carrying capacity to meet the elevated energy demands (Roberts et al. 2005; Berman et al. 2023). Despite evidence that cold temperatures enhance haematological traits in temperate birds (Swanson 2010), we found lower haemoglobin concentrations in the cold (dry) season. This may be because the average minimum temperature at the coldest location is about 4°C, with an average daily temperature of 12°C in the coldest months, which may not be cold enough to drive a strong haematological response (Mlenga et al. 2019). In Addition, the longer summer days of the wet season may have allowed for extended periods of activity, which could be more energetically costly than the longer rest hours in winter (Pokrovsky et al. 2021).

However, territoriality on its own did not have a strong influence on haemoglobin concentration even for strongly territorial birds. We initially hypothesised that territoriality would affect haemoglobin levels due to the increased physical exertion and stress associated with defending territories. However, this was not the case; in fact, though not significant, haemoglobin concentration was lower in territorial birds, possibly reflecting reduced energy expenditure associated with a localised lifestyle and the availability of food resources in their territories (Ord 2021; Salas et al. 2022). In contrast, nonterritorial birds are often more mobile and disperse over large areas (Viera et al. 2011), which requires more energy, equalising energy needs between these ecological groups. Additionally, haemoglobin concentration was significantly higher in aerial foraging birds compared to terrestrial, perching, and generalist species, likely due to the energy‐intensive nature of aerial activities such as prolonged flight, correlated to aerobic power, which in turn appears to be correlated with haemoglobin concentration.

Though not significant, migratory species exhibit slightly higher haemoglobin concentrations, suggesting potential adaptations to the energetic demands of long‐distance migration. However, we failed to detect a significant difference, which may be due to the smaller sample size of migratory birds in our dataset, or potentially a reduction in haemoglobin after migratory birds have settled for some time at the breeding or wintering ground. Nonetheless, this result was unexpected, as it contradicts several studies that demonstrate the significant role of migration in influencing haemoglobin concentration in birds (Barve et al. 2016; Hahn et al. 2022; Yap et al. 2019).

The negative correlation observed between haemoglobin concentration and body mass indicated that smaller birds had higher haemoglobin concentrations, consistent with other studies (Minias 2020; Lill et al. 2013). This may be due to the higher metabolic rates of smaller birds relative to their body size, necessitating higher haemoglobin levels to sustain their elevated energy expenditure (McNab 2015; Londoño et al. 2015). Additionally, short flights that are common among smaller birds are reportedly more energetically costly than steady‐state flights, potentially leading to physiological adaptations that result in higher haemoglobin concentrations (Nudds and Bryant 2000; Klaassen 1996). We also observed a positive association between hand–wing index and haemoglobin concentration that could be attributed to the benefits of efficient flight morphology. Birds with higher hand–wing indices are associated with greater flight capability and mobility; as a result, hand–wing index has been used as a proxy for avian flight efficiency and dispersal ability (Sheard et al. 2020; Weeks et al. 2022; Arango et al. 2022). Hence, birds with larger hand–wing indices have higher haemoglobin concentrations to meet the increased energy expenditure associated with sustained flight.

In the second analysis, we found that the interaction between elevation and most of the modelled ecological traits identified as important in the first analysis, that is, body size, hand–wing index and season were not significant. This means that while these traits were found to have significant relationships with haemoglobin concentration, they do not affect how species respond to changes in elevation. In other words, regardless of differences in body size, flight efficiency, or seasonal changes, birds exhibited broadly similar patterns of haemoglobin–elevation response; they all increase their haemoglobin at a similar rate. Despite elevation and season being observed to be the most critical predictors of haemoglobin concentration in this dataset, the interaction between elevation and season was not statistically significant (pMCMC = 0.074), suggesting that birds maintain consistent physiological adaptations to increasing elevation regardless of seasonal changes. A possible explanation for this observation is that our dataset was restricted to medium elevation (1500 m asl); the increase in haemoglobin concentration required at this elevation remains well within the birds' physiological limits. That is, even though birds in the wet season were observed to generally have higher haemoglobin concentration than in the dry season, in both seasons, birds will still have the same physiological flexibility to further increase haemoglobin concentration in response to increasing elevation. Perhaps, at much higher elevations, where oxygen availability is critically low, differences in the response to increasing elevation may start showing. At those extreme elevations, birds may approach physiological limits, making it more difficult to further increase haemoglobin concentration, resulting in a steeper slope in the haemoglobin–elevation relationship for different ecological groups as oxygen deprivation becomes more extreme.

However, we observed a significant interaction between primary lifestyle and elevation, suggesting that birds with different lifestyles respond differently to the elevational gradient. This may be because, in our dataset, aerial birds primarily belonged to the Hirundinidae family, most of which are aerial insectivores. Their high energy demands for sustained flight likely drive physiological adaptations that support long‐distance movement, potentially influencing their haemoglobin levels (Zhang et al. 2021). They may have adaptations such as increased lung capacity, enhanced oxygen diffusion efficiency, and cardiovascular modifications that allow them to meet high metabolic demands in flight; therefore, instead of relying primarily on haemoglobin adjustments to cope with hypoxia (Scott and Milsom 2009; Butler 2016; Parr et al. 2019). In contrast, terrestrial and perching species are generally more sedentary and localised, often restricted to specific habitat ranges. Unlike highly mobile aerial species, these birds experience more pronounced physiological impacts when exposed to hypoxia, as they may lack alternative mechanisms to compensate for reduced oxygen availability. As a result, even small reductions in oxygen partial pressure with elevation can lead to a significant increase in haemoglobin concentration.

Results from this study indicates that species have evolved specific adaptation mechanisms to meet the unique demands of their ecological niches. The complex interplay between a species' ecology and its physiological response to hypoxia provides valuable insights into the evolutionary strategies that enable bird to thrive in some of the planet's most challenging environments. Explaining why physiological adaptations of species are not universal; even when subjected to the same stressor, some species will be more tolerant than others. This variability in tolerance helps to explain species distribution, particularly why some species cannot survive at higher elevations.

## 5 Conclusion

This study explores the ecological factors shaping haemoglobin concentration in birds. Our findings show that elevation is an important driver of haemoglobin concentration in birds, but other species‐specific traits also play a key role in determining haemoglobin levels among birds in the same area. Species‐specific traits impose different metabolic and oxygen demands, which can influence a species' starting haemoglobin concentration. However, these demands do not generally appear to affect how haemoglobin responds to increasing elevation. Birds at the same elevation had varying haemoglobin concentrations, yet all species increased haemoglobin at a similar rate when exposed to hypoxia, regardless of season or body size, for example. Species with inherently higher haemoglobin concentrations did not increase it less, nor did those with lower haemoglobin increase it more, suggesting that haemoglobin adjustments to hypoxia occur independently of baseline oxygen requirements, at least at moderate elevations, where haemoglobin adjustments likely remain within birds' physiological limits; this pattern holds. This suggests that birds likely compensate for oxygen demands through other adaptation mechanisms other than altering haemoglobin. These findings provide insights into how birds regulate oxygen transport within ecological constraints, contributing to a broader understanding of physiological plasticity in response to environmental gradients.

## Author Contributions

A.M. conceived the ideas and A.M., S.P., K.B., S.A.K. and Z.D.B. designed the study and methodology; Z.D.B. collected the data; Z.D.B. and A.M. analysed the data; Z.D.B. and A.M. led the writing of the manuscript. All authors provided substantial input on various draft versions and gave final approval for publication.

## Ethics Statement

All necessary approvals and methods clearance to conduct this study were obtained from Eswatini Big Game Parks, PI1202.

## Conflicts of Interest

The authors declare no conflicts of interest.

## Statement on Inclusion

Our study brings together researchers with diverse social and academic backgrounds in ecology, molecular biology and behavioural ecology, which strengthens the scope of our research. The first and last authors are from the Global South. By developing a meaningful collaboration with partners from the Global North, we attempt to address the challenges faced by African ecologists.

## Supporting information


Appendix S1.

## Data Availability

All the required data are uploaded as Supporting Information.
