# Supplementary material for: Birds With Distinct Ecological Traits Show Varied Haemoglobin Adaptations Along Elevation Gradients
Source: Ecol Evol. 2025 Apr 9;15(4):e71203. doi: 10.1002/ece3.71203 (PMC11981876; doi:10.1002/ece3.71203)
Supplement: Supplementary file 1 — Appendix S1. [file ECE3-15-e71203-s001.docx]

**Supplementary Information**

**Table S1:** Descriptions of response variables modelled in the study. The column variable, list variables, description describes the variables as used in the study, Variable Type: lists variables as discreate or continuous. Sources; lists data sources of each variable.

| **Variable** | **Description** | **Variable Types** | **Sources** |
| --- | --- | --- | --- |
| Elevation | Height above sea level, recorded at every netting site of this study using a handheld GPS (eTrex 30x, Garmin) | continuous | Measured in the study |
| Season | **Dry**: sampled during the dry season before rainfalls stated  **Wet**: sampled during the wet season after rainfalls begun | discrete | Measured in the study |
| Bird size | Body size measured as body mass (g) | continuous | Measured in the study |
| Hand-Wing Index | 100*DK/Lw - where DK is Kipp’s distance and Lw is wing length (i.e., Kipp’s distance corrected for wing size) | continuous | AVONET database  set (Tobias et al., 2022) |
| Migration | **Sedentary** – Migration data was obtained from BirdLife with ‘full migrants’ scored here as migratory and all others (partial migrants, altitudinal migrants, non-migrants, and nomads). **Migratory** –Majority of population undertakes long-distance migration | categorical | AVONET database  set (Tobias et al., 2022) |
| Primary lifestyle | **Aerial** - species spends much of the time in flight, and hunts or forages predominantly on the wing;  **Terrestrial** - species spends majority of its time on the ground, where it obtains food while either walking **Perching** - species spends much of the time perching above the ground, either in branches of trees and other vegetation (i.e. arboreal), or on other raised substrates including rocks, buildings, posts, and wires;  **Generalist** - species has no primary lifestyle because it spends time in different lifestyle classes | categorical | AVONET database  set (Tobias et al., 2022) |
| Territoriality | **Non-territorial** - species lack any form of territorial behaviour  **Weakly territorial** - species only defend seasonal territories such as nesting sites and mating display sites  **Strongly territorial**- species defended territories all year round | categorical | Sheard et al., 2020 |
